# Two subtypes of major depressive disorder are identified from individualized gray matter morphological abnormalities in a large multi-site dataset

**DOI:** 10.1017/S0033291725101499

**Published:** 2025-09-01

**Authors:** Keke Fang, Baohong Wen, Liang Liu, Ya Tian, Huiting Yang, Shaoqiang Han, Xianfu Sun, Lianjie Niu

**Affiliations:** 1 Department of Pharmacy, The Affiliated Cancer Hospital of Zhengzhou University and Henan Cancer Hospital; 2 Henan Engineering Research Center for Tumor Precision Medicine and Comprehensive Evaluation, Henan Cancer Hospital; 3 Henan Provincial Key Laboratory of Anticancer Drug Research, Henan Cancer Hospital; 4 Department of Magnetic Resonance Imaging, The First Affiliated Hospital of Zhengzhou University, Henan Province, China; 5 Department of Breast Disease, Henan Breast Cancer Center, The Affiliated Cancer Hospital of Zhengzhou University and Henan Cancer Hospital

**Keywords:** MDD subtype, individualized, gray matter volume, normative modelling

## Abstract

**Background:**

Neuroimaging studies provide compelling evidence that major depressive disorder (MDD) is associated with widespread gray matter morphological abnormalities. However, significant interindividual variability complicates the interpretation of group-level findings, highlighting the need for investigating potential MDD subtypes.

**Methods:**

In this study, we aimed to identify subtypes of MDD based on individualized deviations from normative gray matter volumes (GMVs), as estimated using a normative model derived from healthy controls (HCs). We leveraged a large, multi-site dataset of high-resolution structural MRI scans, comprising 1,276 MDD patients and 1,104 matched HCs. To explore the transcriptional and molecular mechanisms underlying the observed structural abnormalities, we examined the relationships between GMV deviations, transcriptomic similarities (as measured by the correlated gene expression [CGE] connectome), and the distribution of neurotransmitter receptors/transporters.

**Results:**

Our results revealed two reproducible MDD subtypes, each exhibiting distinct patterns of GMV abnormalities across study sites. Subtype 1 displayed increased GMVs in cerebral regions and decreased GMVs in cerebellar regions, whereas subtype 2 showed the opposite pattern, with decreased GMVs in cerebral regions and increased GMVs in cerebellar areas. The identified GMV abnormalities were differentially associated with neurotransmitter receptor/transporter distributions. Furthermore, these abnormalities were linked to transcriptionally connected gene networks, suggesting genetic underpinnings for both subtypes. Notably, the two subtypes exhibited distinct CGE-informed disease epicenters.

**Conclusions:**

This study identifies two robust MDD subtypes, providing new insights into the neurobiological and genetic bases of MDD and offering a potential advancement in the nosology of the disorder.

## Introduction

Major depressive disorder is one of the leading causes of disability globally, affecting approximately 350 million people annually (Murray et al., 2012; Schmaal et al., 2017a). Despite extensive research, understanding the underlying mechanisms of MDD remains challenging due to significant heterogeneity among individuals with the same diagnosis (Beijers, Wardenaar, van Loo, & Schoevers, 2019b). Two individuals with MDD may present with vastly different symptom profiles, course trajectories, and treatment responses (Hasler, 2010; Krishnan & Nestler, 2008). This considerable variability supports the notion that MDD encompasses multiple subtypes, though their neurobiological foundations are still not well understood, impeding the development of precise clinical guidelines. Clinically, MDD patients are often categorized into distinct subtypes based on symptom presentation (Lynch, Gunning, & Liston, 2020). However, such symptom-based classifications have not led to effective, evidence-based treatment options. MDD patients with similar symptoms may still harbor different biological mechanisms, limiting the utility of these categories for differentiating etiologically distinct groups.

An alternative approach is to identify neurophysiological subtypes through clustering individuals based on objective neuroimaging signatures (Clementz et al., 2016; Drysdale et al., 2017). This method has begun to shed light on how diverse biological mechanisms may contribute to the overlapping and heterogeneous clinical manifestations of psychiatric disorders (Clementz et al., 2016; Hill, Reilly, Keefe, Gold, & Sweeney, 2013). Structural neuroimaging studies using magnetic resonance imaging (MRI) have consistently reported abnormalities in cortical and subcortical regions, including the orbitofrontal cortex, superior frontal cortex, and hippocampus (Li et al., 2020; Schmaal et al., 2017b; Schmaal et al., 2016). However, findings across studies have been inconsistent, largely due to the predominant use of case–control designs that overlook the interindividual heterogeneity within MDD (Belleau, Treadway, & Pizzagalli, 2019; Bora, Fornito, Pantelis, & Yücel, 2012; Chen et al., 2016; Treadway et al., 2015). Contradictory and even opposing patterns of structural alterations have been observed in key regions such as the insula and anterior cingulate cortex (Peng et al., 2015; Schmaal et al., 2017b; Zorlu, Cropley, Zorlu, Delibas, & Pantelis, 2017). These heterogeneous alterations may reflect distinct subtypes of MDD (Feder et al., 2017; Wang et al., 2021). Recently, the normative model has been proposed as a method to capture individual variability in structural abnormalities within psychiatric disorders (Marquand, Rezek, Buitelaar, & Beckmann, 2016; Wolfers et al., 2018). Similar to growth charts in pediatric medicine, which map child development based on height or weight, the normative model maps clinically relevant biological measures – such as neuroimaging metrics – using healthy control data (Marquand et al., 2016; Wolfers et al., 2018). Deviations from these normative models in brain structure and function show promise in identifying subtypes of psychiatric disorders, including MDD (Li et al., 2024a; Liu et al., 2024; Sun et al., 2023a). However, most studies to date have been limited by small sample sizes and single-site designs, leaving open the question of whether individualized deviations from normative models of gray matter volume can identify robust and reproducible subtypes in multi-site neuroimaging datasets.

MDD arises from complex interactions across biological systems, spanning genes, molecules, cells, networks, and behavior (Anderson, Collins, Kong, Fang, & Holmes, 2020). Increasing evidence suggests a strong relationship between gray matter morphological abnormalities and the brain’s genetic architecture and neurotransmitter receptor profiles. For instance, recent studies have demonstrated that gene expression profiles across the brain are closely associated with macroscopic neuroimaging phenotypes in healthy individuals (Krienen, Yeo, Ge, Buckner, & Sherwood, 2016; Li & Seidlitz, 2021; Vértes et al., 2016) and altered neuroimaging metrics in brain disorders, as evidenced by the Allen Human Brain Atlas (AHBA), which provides gene expression data from six postmortem adult brains (Han et al., 2022b; Hawrylycz et al., 2012; Morgan, Seidlitz, & Whitaker, 2019; Romme, de Reus, Ophoff, Kahn, & van den Heuvel, 2017). Transcriptional similarity between brain regions – referred to as the correlated gene expression (CGE) connectome – correlates with structural and functional brain networks (Arnatkeviciute, Fulcher, Oldham, Tiego, & Fornito, 2021; Fulcher & Fornito, 2016). Recent study by Li et al. has shown that cortical thickness differences in MDD subtypes are constrained by CGE, with these subtypes exhibiting distinct CGE-informed epicenters, providing new evidence for a connectome-based spreading process in mental disorders (Hansen et al., 2022c; Li et al., 2024a; Yang et al., 2021b). Additionally, neurotransmitter receptor profiles contribute to cortical morphology across various brain disorders (Hansen et al., 2022c). Understanding the transcriptional and molecular basis of gray matter abnormalities in MDD subtypes may enhance our understanding of their pathophysiology and help guide treatment strategies (Chen et al., 2021).

In this study, we aimed to identify potential subtypes of MDD based on individualized deviations from a normative model of gray matter volume in a large, multi-site cohort consisting of 1,276 MDD patients and 1,104 healthy controls (HCs) across 24 research sites. We first defined individualized deviations by comparing regional gray matter volumes in each patient to normative estimates derived from HCs. MDD subtypes were then identified using a K-means clustering algorithm and characterized in terms of clinical variables and voxel-wise gray matter abnormalities relative to HCs. Next, we predicted regional gray matter abnormalities based on deviations in neighboring regions as defined by the CGE connectome and explored potential epicenters within each MDD subtype. Finally, to gain a deeper understanding of the molecular mechanisms underlying these structural abnormalities, we examined the associations between the identified gray matter alterations and the distribution of neurotransmitter receptors.

## Materials and methods

### Imaging dataset and preprocessing

The MDD imaging datasets used in this study were sourced from the REST-meta-MDD consortium (http://rfmri.org/REST-meta-MDD) (Chen et al., 2022; Yan et al., 2019). The dataset includes data from 24 research sites, comprising 1,276 patients diagnosed with MDD (mean age: 36.23 ± 21.38 years; females: 63.71%) and 1,104 healthy controls (HCs) (mean age: 36.15 ± 24.55 years; females: 58.06%). All data collection procedures were approved by the local ethics committees at each research site. MDD diagnoses were made by experienced psychiatrists based on DSM-IV criteria. Depression severity in patients was assessed using the Hamilton Depression Rating Scale (HAMD) (Hamilton, 1960). Among the MDD patients, 538 were first-episode, 282 were recurrent, 447 were medication-naive, and 408 were on medication. Further details on demographic characteristics, clinical assessments, and imaging acquisition protocols have been previously described (Chen et al., 2022; Yan et al., 2019).

Structural MRI data were preprocessed following standardized protocols across sites to minimize analytic variation. The specific preprocessing steps for structural MRI data are detailed in prior publications (Chen et al., 2022; Yan et al., 2019). For this study, we utilized gray matter volume (GMV) maps derived from voxel-based morphometry (VBM) analysis, which were made available by the REST-meta-MDD consortium (http://rfmri.org/REST-meta-MDD) (Ashburner, 2009; Craddock, James, Holtzheimer, Hu, & Mayberg, 2012).

### Normative model of gray matter volume and individualized gray matter morphological abnormalities

To mitigate non-biological variance introduced by differences in scanners and acquisition protocols, we applied the Combat method to harmonize GMV data across different sites. Combat has been demonstrated to effectively remove site-related variance while preserving biological variability in neuroimaging studies (Jean-Philippe et al., 2017.

We then constructed a normative model of GMV. In line with methods outlined in previous research (Marquand et al., 2016; Wolfers et al., 2018), we trained a Gaussian process regression model to estimate the normative range of regional GMV values based on age and sex in HCs, using the Shen 268 brain atlas (Shen, Tokoglu, Papademetris, & Constable, 2013). This model was then applied to the MDD patient group. Individualized GMV abnormalities were represented as Z-scores, with a positive Z-score indicating a GMV value higher than the HC average, and a negative Z-score indicating a lower GMV value.

Before applying the model to the patient group, we assessed its performance using the following validation strategies: (1) 10-fold cross-validation, repeated 100 times; (2) leave-one-site-out cross-validation, where one site was used as the test set, and the remaining sites served as the training set in turn. Model performance was evaluated by calculating the standardized mean squared error (MSE) between the true and predicted GMV values (Marquand et al., 2016). After confirming the model’s validity, Z-scores were computed for each patient relative to the HCs, resulting in a Z-score matrix (patients × brain regions, N × 268).

To further investigate the intersubject heterogeneity of extreme deviations, we generated a spatial overlap map. This map quantified the percentage of participants exhibiting extreme deviations (defined as Z-scores exceeding ±1.96, representing a two-tailed 95% confidence interval). This approach allowed us to delineate extreme positive or negative deviations from the normative model (Haas et al., 2024; Liu et al., 2024; Sun, Sun, Lu, et al., 2023a).

### Identifying MDD subtypes using individualized gray matter morphological abnormalities

We next sought to identify potential MDD subtypes based on individualized gray matter morphological abnormalities. To assess the significance of clustering, we first applied the SigClust approach. This method tests the hypothesis that the data fit a single Gaussian distribution, indicating no subtypes (Dinga et al., 2019). Upon confirming the presence of subtypes, the K-means algorithm was conducted to identify MDD subtypes based on individualized gray matter morphological abnormalities, using the correlation distance (one minus the correlation coefficients between samples) as the distance metric. To avoid local minima, K-means was run 100 times with different centroid initializations (Allen et al., 2014). The optimal number of subtypes, ranging from 2 to 10, was determined using a cluster ensemble voting technique, which incorporates 26 indices to ensure an unbiased selection of the optimal subtype (Charrad, Ghazzali, Boiteau, & Niknafs, 2015).

Cluster stability was evaluated using two validation methods: subsample validation and leave-one-site-out validation. In the first approach, to minimize the influence of any small subset of patients, 90% of the participants were randomly selected, and k-means clustering was performed on this subsample. The Adjusted Rand Index (ARI) was calculated to compare the clustering results from the subsample with those derived from the full sample, assessing the consistency across iterations. This process was repeated 100 times to ensure robustness. In the second method, K-means clustering was performed excluding one site at a time, and the ARI between the clustering results obtained from each leave-one-site-out approach and the full-site clustering was calculated.

### Characterizing subtypes

The identified subtypes of MDD were characterized based on demographic and clinical variables including sex, age, illness duration, symptom severity, and the distribution of first-episode versus recurrent, and untreated versus treated patients. Statistical comparisons were made using appropriate tests, including *t*-tests for continuous variables and chi-square tests for categorical variables.

Voxel-wise gray matter morphological differences between each MDD subtype and HCs were assessed using two-sample *t*-tests in SPM12 (https://www.fil.ion.ucl.ac.uk/spm/software/spm12/), with sex, age, and site as covariates. Additionally, voxel-wise gray matter abnormalities for the entire patient cohort were compared with HCs. Statistical significance was set at *p* < 0.05 with Bonferroni correction (family wise error *p*_FWE_ < 0.05).

### Associations between neurotransmitter receptors/transporters and gray matter morphological abnormalities of the identified subtypes

We examined the relationships between neurotransmitter receptors/transporters and gray matter morphological abnormalities of the identified subtypes. The data on neurotransmitter receptors/transporters were sourced from the PET-derived atlas compiled by Hansen et al. (Hansen & Shafiei, 2022b). This atlas includes several receptors and transporters: serotonin (5HT_1A_ [Savli et al., 2012], 5HT_1B_ [Baldassarri et al., 2018; Gallezot et al., 2010; Matuskey et al., 2014; Murrough et al., 2011a; Murrough et al., 2011b; Saricicek et al., 2015; Savli et al., 2012], 5HT_2A_ [Beliveau & Ganz, 2017], 5HT_4_ [Beliveau & Ganz, 2017], 5HT_6_ [Radhakrishnan et al., 2020; Radhakrishnan et al., 2018], 5HTT [Beliveau & Ganz, 2017]), norepinephrine (α_4_β_2_ [Baldassarri et al., 2018; Hillmer et al., 2016], M_1_ [Naganawa et al., 2021], VAChT [Aghourian, Legault-Denis, Soucy, & Rosa-Neto, 2017; Bedard et al., 2019]), cannabinoid (CB_1_ [D’Souza et al., 2016; Neumeister et al., 2012; Normandin et al., 2015; Ranganathan et al., 2016]), dopamine (D_1_ [Kaller et al., 2017], D_2_ [Sandiego et al., 2015; Slifstein et al., 2015; Smith et al., 2019; Zakiniaeiz et al., 2019], DAT [Dukart et al., 2018]), GABA (GABA_a_ [Nørgaard et al., 2021]), histamine (H_3_ [Gallezot et al., 2017], glutamate (mGluR_5_ [DuBois et al., 2016; Smart et al., 2019], NMDA [Galovic et al., 2021; McGinnity et al., 2014], opioid (MOR [Kantonen et al., 2020]), and norepinephrine (NET [Belfort-DeAguiar et al., 2018; Ding et al., 2010; Li et al., 2014; Sanchez-Rangel et al., 2020]). PET images from each study were averaged across participants, normalized to the MNI-ICBM 152 nonlinear 2009 template, and subsequently parcellated into 268 brain regions. The average receptor/transport densities were Z-scored (Hansen & Shafiei, 2022a, 2022b). To assess the regional gray matter morphological abnormalities, unthresholded t-statistics for each region were averaged. A multilinear regression model was constructed for each subtype, incorporating neurotransmitter receptor/transport profiles and regional gray matter abnormalities. The statistical significance of these models was determined through permutation testing (10,000 iterations), with Bonferroni correction applied to adjust for multiple comparisons.

Subsequently, a dominance analysis was conducted for each model to evaluate the relative contribution of each predictor (i.e., neurotransmitter receptor/transport profiles) to the overall model fit (Budescu & David, 1993). Dominance analysis estimates the importance of predictors by applying the multilinear model across all possible combinations of predictors. In this study, the total dominance value of each predictor was derived from the average increase in explained variance (R^2^) when it was added to the sub-models (Azen & Budescu, 2003; Budescu & David, 1993; Hansen & Shafiei, 2022a).

### Disease epicenter of correlated gene expression connectome for each subtype

To explore the genetic underpinnings of gray matter morphological abnormalities in each subtype, we analyzed the associations between the CGE connectome and regional gray matter morphology. The CGE connectome was derived from regional gene expression data obtained from the AHBA (http://human.brain-map.org) (Hawrylycz et al., 2012). The raw expression data were preprocessed following established protocols (Fang, Hou, Niu, Han, & Zhang, 2024; Hawrylycz et al., 2012) and transcriptome similarities were quantified using Pearson’s correlation coefficients between regional gene expressions, resulting in a symmetric CGE connectome (268 **×** 268). Only positive correlations in the CGE connectome were retained for further analysis.

The relationship between the CGE connectome and regional gray matter abnormalities was assessed using methods previously outlined in the literature (Shafiei et al., 2020a). For each subtype, we calculated the normalized collective differences of transcriptomic neighbors for each region ( 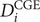 ) as described in the following:

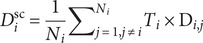

where 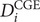 represents the normalized collective abnormalities of the transcriptomic neighbors for region *i*, 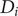 is the regional abnormalities (unthresholded t-statistic) of region *i*, 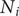 is the number of neighboring regions with transcriptomic connections, and 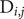 is the strength of the transcriptomic connection between region *i* and *j.* For each subtype, the 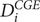 was predicted based on the regional abnormalities of neighboring regions. The Pearson’s correlation coefficient between true abnormalities and 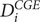 across all brain regions was then calculated. To assess whether gray matter abnormalities specific to the identified subtypes exhibited a stronger association with the CGE connectome compared to all patients, we conducted a similar analysis for the entire patient cohort. The Pearson’s correlation coefficients were compared using Steiger’s *Z* test (Feng et al., 2018; Fang et al., 2021).

Next, we identified potential disease epicenters for each subtype. A brain region was considered a disease epicenter if, along with its connected neighbors, it showed significantly greater abnormalities than other regions (Shafiei et al., 2020a). Brain regions were ranked based on the differences in unthresholded *t*-statistics and CGE-informed abnormalities ( 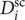 ) in ascending order. The average ranking values were then used to determine the disease epicenter likelihood rankings, with statistical significance evaluated through permutation testing (10,000 iterations). A brain region was designated as a disease epicenter if its likelihood ranking was significantly higher than expected by chance (permutation *p* < 0.01).

### Reproducibility analysis

To ensure the robustness of our findings and assess the impact of brain parcellation choices, we validated our main results using an alternative brain atlas, the Automated Anatomical Labeling (AAL) atlas, which operates at a different resolution (Tzourio-Mazoyer et al., 2002).

## Results

### Clinical demographics

The clinical and demographic information for the datasets utilized in this study is presented in Supplementary Table S1.

### Heterogeneous individualized extreme deviations in patients with MDD

The results of the 10-fold cross-validation and leave-one-site-out cross-validation demonstrated the high generalizability of the normal models of GMVs in predicting GMVs in HCs. The mean MSE for the 10-fold and leave-one-site-out cross-validation was 0.73 (±0.08) and 0.82 (±0.09), respectively. The spatial distributions of the MSE are shown in Supplementary Figure S1.

**Figure 1.**
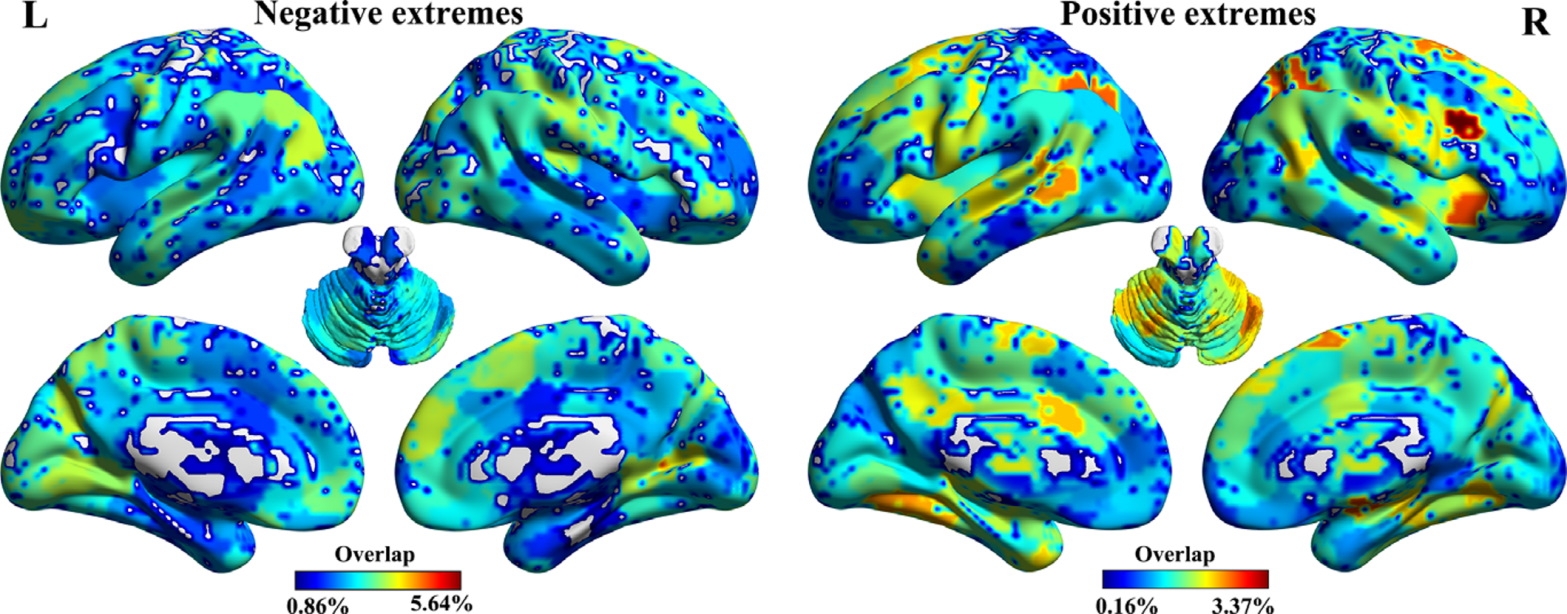
Spatial overlap maps of extreme deviations in MDD patients. The left panel illustrates the overlap of extreme negative deviations (Z-scores <−1.96), and the right panel shows the overlap of extreme positive deviations (Z-scores >1.96) across MDD patients.

After confirming the performance of the normative model, we applied the trained model derived from HCs to patients with MDD to identify individualized gray matter morphological abnormalities. MDD patients exhibited heterogeneous patterns of abnormal gray matter morphology. Specifically, 59.01% (*n* = 753) of MDD patients showed significant negative deviations from the normative model in at least one brain region (Figure 1). These negative deviations were primarily found in the superior frontal gyrus, calcarine, dorsolateral prefrontal cortex (DLPFC), superior parietal cortex, and middle occipital cortex (Figure 1). Conversely, 50.71% (*n* = 647) of MDD patients showed extreme positive deviations in at least one brain region (Figure 1), most notably in the right inferior frontal gyrus, right amygdala, hippocampus, superior parietal cortex, and middle occipital cortex (Figure 1). Notably, the percentage of patients exhibiting extreme deviations from the normative range in any single brain region was relatively low, with the most common extreme deviation shared by only 5.64% of patients (Figure 1). These findings highlight substantial heterogeneity in individualized deviations from the normative model among MDD patients, underscoring the need to identify more homogeneous MDD subtypes.

### Two subtypes manifesting opposite patterns of gray matter morphological abnormalities are identified

**Figure 2.**
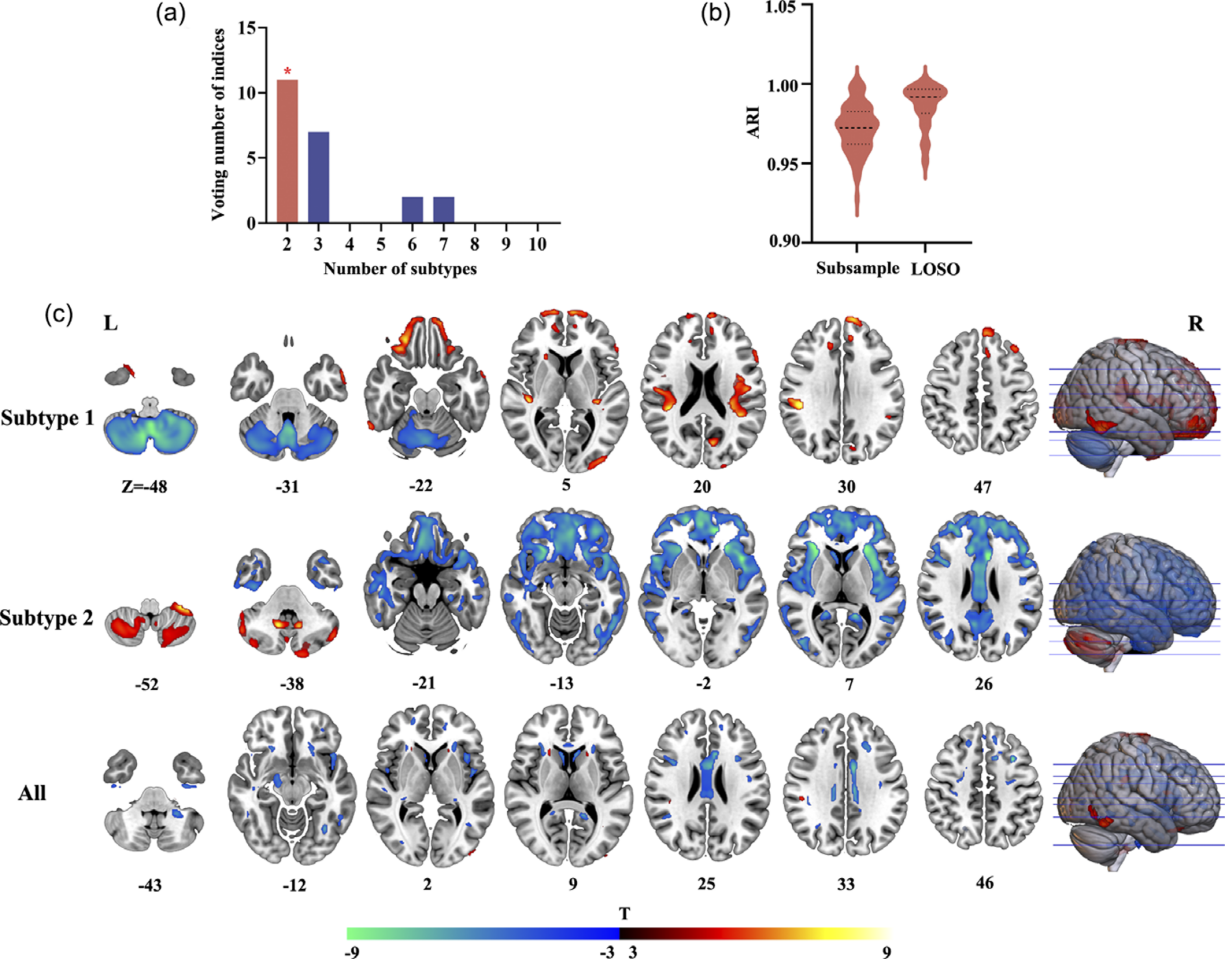
Subtyping results. (a) The optimal two-cluster solution is identified by the cluster ensemble voting technique (marked by a red asterisk). (b) Subsample validation and leave-one-site-out (LOSO) validation results. The adjusted Rand Index (ARI) values between clustering outcomes from validation subsamples and the main results are shown. (c) Voxel-wise gray matter abnormalities in the identified subtypes relative to healthy controls.

Significant cluster testing confirmed the existence of distinct MDD subtypes characterized by individualized gray matter abnormalities (Supplementary Figure S2). The cluster ensemble voting technique identified a two-cluster solution as optimal for the entire MDD sample (Figure 2a). Patients were subsequently classified into two subtypes via K-means clustering (Subtype 1, *N* = 615; Subtype 2, *N* = 661). Subsample validation and leave-one-site-out validation results confirmed the robustness of these clustering outcomes, with the mean adjusted Rand index (ARI) between validation results and the primary results being 0.97 (±0.01) and 0.99 (±0.01), respectively. The distributions of ARI values are shown in Figure 2b.

Voxel-wise analysis of gray matter morphology revealed that the two subtypes displayed opposing patterns of deviations from HC GMVs with spatial Pearson’s correlation of −0.36 (*p* < 0.01). Specifically, subtype 1 showed significantly increased GMVs in regions such as the inferior and superior frontal gyrus, anterior cingulate cortex, insula, superior temporal gyrus, middle occipital gyrus, caudate, and inferior parietal lobule, while GMVs were decreased in cerebellar regions (Supplementary Table S1 and Figure 2c). In contrast, subtype 2 exhibited decreased GMVs in several brain regions, including the midline frontocortical areas (frontal gyrus, cingulate cortex), insula, and striatum, with increased GMVs in cerebellar regions (Supplementary Table S1 and Figure 2c). Despite these differences, the two subtypes did not show significant variation in clinical and demographic variables, including sex, age, illness duration, symptom severity, and the proportion of first-episode versus recurrent, or untreated versus treated patients (all uncorrected *p* > 0.05).

### Gray matter morphological abnormalities in subtypes exhibit distinct associations with neurotransmitter receptors/transporters

**Figure 3.**
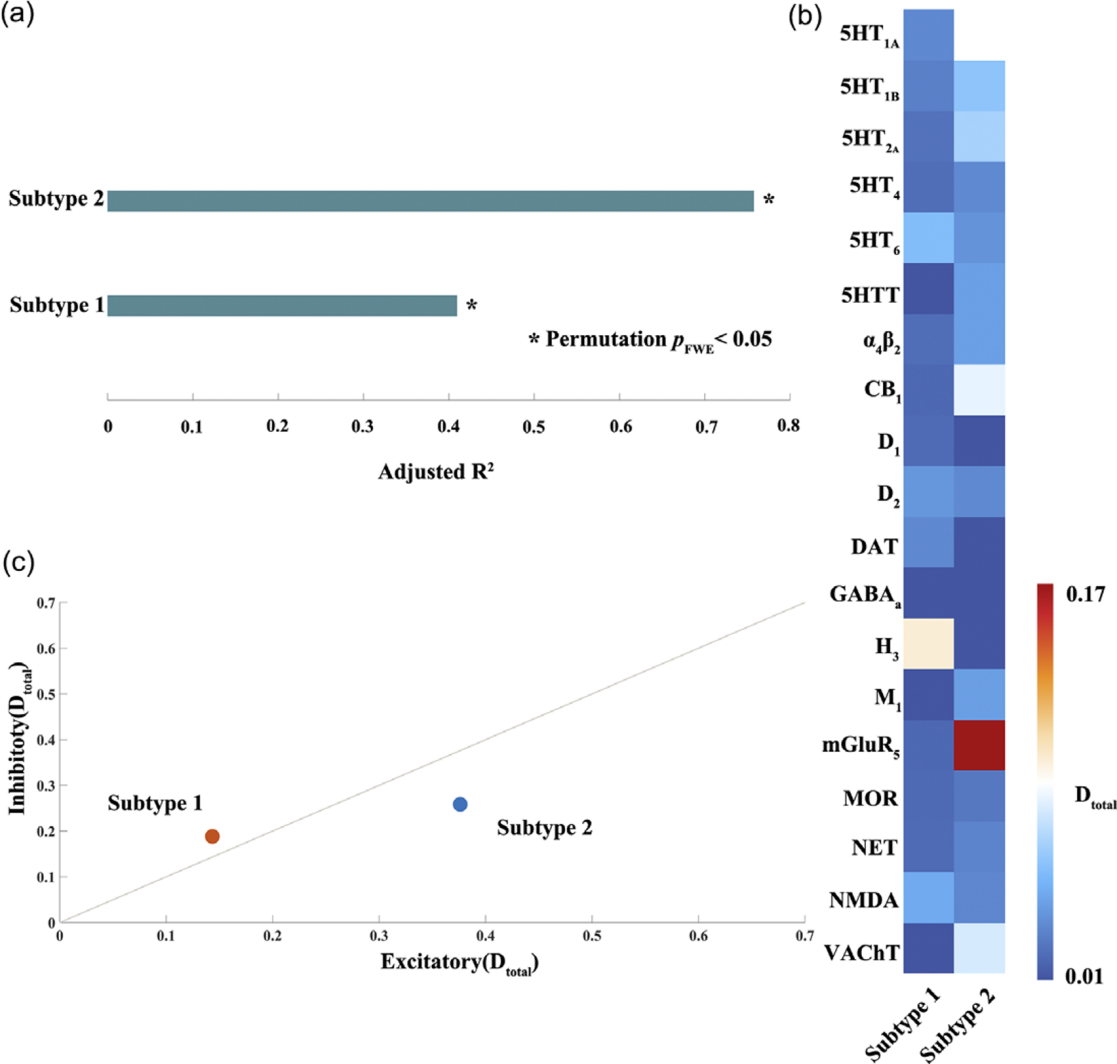
Association between gray matter morphological abnormalities of the identified subtypes and neurotransmitter receptor/transporter distribution. (a) Multilinear models assessing the association between neurotransmitter receptors/transporters and gray matter abnormalities of subtypes. The bar plot illustrates the goodness-of-fit (adjusted *R* ^2^) for each model, with Bonferroni correction (*p*_FWE_ < 0.01). (b) Dominance analysis for determining the relative importance of predictors in each model. (c) Cumulative contributions of excitatory versus inhibitory receptors to gray matter abnormalities in the identified subtypes, with the grey line representing the identity line.

We further explored the associations between neurotransmitter receptors/transporters and gray matter morphological abnormalities within each subtype. This was accomplished by constructing multilinear models to account for the spatial distributions of receptors/transporters and the differential patterns of gray matter abnormalities. The goodness-of-fit (adjusted *R* ^2^) of these models was 0.88 (*F*-statistic (268,248) = 101.00, *p*_FWE < 0.01) for subtype 1 and 0.90 (*F*-statistic (268,248) = 128.00, *p*_FWE < 0.01) for subtype 2, indicating strong model performance (see Figure 3a). Dominance analysis revealed that specific neurotransmitter receptors were crucial for each subtype. Notably, H3 receptor was significant for subtype 1, while mGluR5 was prominent for subtype 2 (Figure 3b).

Furthermore, we categorized receptors into excitatory and inhibitory types and calculated their cumulative contributions to gray matter abnormalities. Our results indicated that the gray matter abnormalities in subtype 1 were primarily associated with excitatory receptors, while those in subtype 2 were predominantly linked to inhibitory receptors (Figure 3c).

### Divergent CGE-informed disease epicenters in the identified subtypes

**Figure 4.**
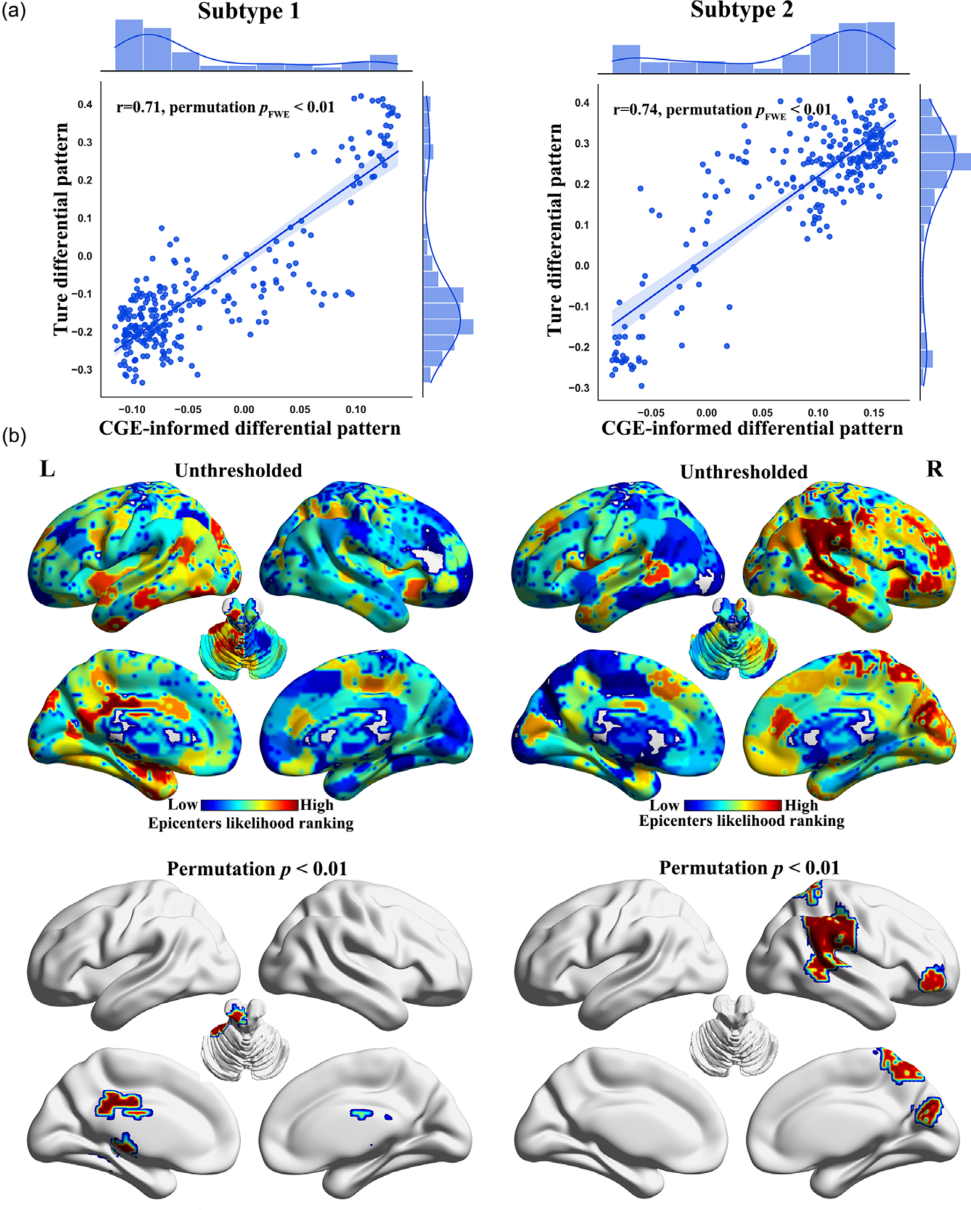
Association between gray matter abnormalities in the identified subtypes and transcriptomic similarity based on correlated gene expression (CGE) connectome. (a) Pearson’s correlation coefficient between the regional patterns of gray matter abnormalities and the CGE-informed differential pattern. (b) Putative disease epicenters for each subtype.

Both subtypes exhibited significant correlations between regional gray matter abnormalities and the regional differences in connected neighbors within the CGE connectome (Subtype 1: *r* = 0.71, *p*_FWE_ < 0.01; Subtype 2: *r* = 0.73, *p*_FWE_ < 0.01; see Figure 4a). These findings suggest that the gray matter abnormalities in both subtypes are influenced by the CGE connectome. Moreover, gray matter abnormalities in the identified subtypes showed significantly higher Pearson’s correlations with the CGE connectome than in the overall MDD patient group (Subtype 1 vs. all patients, Steiger’s *Z* = 5.98, *p*_FWE_ < 0.01; Subtype 2 vs. all patients, Steiger’s *Z* = 6.68, *p*_FWE_ < 0.01).

Distinct disease epicenters were identified for the two subtypes (Figure 4b). Subtype 1 showed epicenters in the cerebellar anterior lobe, hippocampus, thalamus, and posterior cingulate cortex (permutation *p*_FWE_ < 0.01). Subtype 2, however, exhibited epicenters in regions such as the middle frontal gyrus, inferior parietal lobule extending to the superior temporal gyrus and cuneus (permutation *p*_FWE_ < 0.01).

### Reproducibility analysis

To assess the robustness of our findings across different brain parcellation schemes, we validated our main results using the AAL atlas. Significant cluster tests confirmed the presence of MDD subtypes (Supplementary Figure S3), and the cluster ensemble technique again identified the two-cluster solution as optimal for the MDD sample (Supplementary Figure S4A). The ARI values between the original and the AAL-based clustering outcomes were 0.77, indicating high consistency. The gray matter abnormalities of the identified subtypes remained largely unchanged (Supplementary Figure S4B). The spatial Spearman’s correlations between the abnormal patterns from the original and AAL-based analyses were 0.99 (*p*_FWE_ < 0.01) for subtype 1 and 0.99 (*p*_FWE_ < 0.01) for subtype 2, confirming the reproducibility of the identified subtypes.

## Discussion

This study identified two robust and reproducible MDD subtypes based on individualized gray matter morphological abnormalities, derived from a large and multi-site MDD cohort. The identified subtypes show opposite patterns of gray matter morphological abnormalities compared to HCs. Specifically, we observed that subtype 1 is predominantly associated with excitatory receptor abnormalities, while subtype 2 is linked to inhibitory receptor dysfunctions. Moreover, the gray matter morphological abnormalities of the identified subtypes were constrained by CGE connectome and were related to distinct CGE-informed epicenter profiles. These findings contribute to a deeper understanding of the structural brain heterogeneity in MDD and suggest molecular mechanisms that may differentiate these subtypes.

Patients with MDD exhibit substantial individual variability in structural and functional neuroimaging features, including gray matter volume and functional connectome, to the extent that extreme person-specific deviations from normal expectation for regional GMV never exceed 6.21% of cases with MDD (Han et al., 2023a; Li et al., 2024b; Segal et al., 2023; Sun et al., 2023b). This heterogeneity is in line with the clinical heterogeneity, posing significant challenges for understanding the disorder’s etiology. Prior studies have shown inconsistent results regarding structural brain abnormalities, with some contradictory findings (Bora et al., 2012; Chen et al., 2016; Kempton et al., 2011; Schmaal et al., 2016). The identification of homogeneous MDD subtypes is critical. While previous research has used data-driven approaches to classify MDD subtypes based on neuroimaging features, such studies typically relied on machine learning models applied to single, smaller cohorts (Beijers, Wardenaar, van Loo, & Schoevers, 2019a; Chen, Dai, & Lin, 2023; Han et al., 2022a; Xu et al., 2023; Yang et al., 2021a; Yang et al., 2025). In contrast, our approach demonstrates robust subtype identification, validated by subsample and leave-one-site-out validation, which supports the reproducibility of the findings. Furthermore, the two subtypes show opposite patterns of gray matter abnormalities relative to HCs. Specifically, subtype 1 exhibits increased GMVs in cortical regions and decreased GMVs in cerebellar areas, while subtype 2 shows the reverse pattern. A recent study identified similar subtypes based on cortical thickness patterns (Li et al., 2024a), and our results extend these findings to include subcortical and cerebellar regions, areas frequently implicated in depression pathophysiology (Gong & He, 2015; Kaiser, Andrews-Hanna, Wager, & Pizzagalli, 2015; Otte et al., 2016). The cerebellum, in particular, is of interest due to its anatomical connections to limbic regions (Turner et al., 2007) and its critical role in emotion and cognitive processing (Moulton et al., 2011; O’Reilly, Beckmann, Tomassini, Ramnani, & Johansen-Berg, 2010), with interactions between the cerebellum and regions especially the hippocampus being central to depression (Steinke et al., 2017). However, both increased and atrophic cerebellum volume are reported previously (Chen et al., 2024a; Chen et al., 2025; Chen, Sui, Yang, Lv, & Wang, 2024b). Despite prior reports of both enlarged and atrophic cerebellar volumes (Han et al., 2023b), we found no significant differences in clinical variables such as illness duration or symptom severity between the subtypes, suggesting that the brain differences identified are not captured by conventional clinical measures and may have novel clinical implications.

To explore the biological mechanisms underlying the observed gray matter abnormalities, we examined their associations with neurotransmitter receptor profiles. Dysregulation of neurotransmitters is a key factor in the pathophysiology of MDD, and modern antidepressant treatments target specific neurotransmitter systems (Hansen & Shafiei, 2022a). Structural and functional abnormalities in psychiatric disorders have been linked to neurotransmitter receptor profiles (Hansen & Shafiei, 2022a, 2022b), and our findings corroborate this relationship. Specifically, subtype 1 is predominantly associated with the histamine H_3_ receptor, while subtype 2 is linked to the mGluR_5_ receptor. Both receptors have been implicated in MDD pathology; for example, histamine regulates various functions, including learning, memory, and appetite, and H_3_ receptor antagonists have shown pro-cognitive and antidepressant effects (Iida et al., 2017; Femenía et al., 2015). These receptor-specific associations may help explain why traditional antidepressant treatments often have limited efficacy (Möller, 2008), highlighting the potential for more tailored interventions. Our findings provide new insights into the molecular bases underlying structural abnormalities in MDD subtypes, with implications for precision medicine.

Additionally, we find that gray matter abnormalities in the identified subtypes align with the CGE connectome, further supporting a network-based spread of structural abnormalities in MDD. Neuroimaging studies have demonstrated that these abnormalities often follow the underlying brain connectome rather than being randomly distributed, with structural changes originating from disease epicenters and propagating to other brain regions (Shafiei et al., 2020b; Wannan et al., 2019; Zhou, Gennatas, Kramer, Miller, & Seeley, 2012). This network-based hypothesis has been supported in MDD and other psychiatric disorders (Han, Cui, et al., 2023a; Li et al., 2024b). Moreover, gray matter morphology is highly heritable and constrained by genetic factors (Grasby et al., 2020; Panizzon et al., 2009). Our results confirm that gray matter abnormalities are not only influenced by genetic architecture but also constrained by transcriptomic similarity, providing further evidence for the network-based spread of these abnormalities. The distinct epicenter patterns observed in our subtypes suggest divergent pathological trajectories, which could be further validated in longitudinal studies.

This study has several limitations. First, the cross-sectional design raises questions about the stability and generalizability of the identified subtypes. Future research should validate these findings with independent, longitudinal datasets to assess their robustness. Second, the limited clinical data available in this study constrained our ability to explore differences in other clinical variables, such as medication dosage or comorbidities. Future studies incorporating more comprehensive clinical data would provide deeper insights into subtype characteristics and their potential implications for treatment. Third, we did consider other neuroimaging metrics, such as intrinsic brain connectivity, which also provides a potentially mechanistic framework for understanding mental disorders (Cheng et al., 2025; Mo et al., 2024; Xu et al., 2024; Zhang, Xu, Ma, Qian, & Zhu, 2024). Finally, the impact of comorbidities on these results was not examined, and future study should investigate whether comorbid conditions influence the identified subtypes or contribute to additional subtyping within MDD.

In conclusion, we identified two distinct subtypes of MDD based on individualized gray matter morphological abnormalities, which exhibit contrasting patterns relative to the healthy population. These subtypes are associated with different CGE-informed epicenters and show divergent neurotransmitter receptor profiles, pointing to distinct molecular mechanisms underlying their structural brain differences. These findings contribute to our understanding of the heterogeneous nature of MDD and offer potential directions for more personalized therapeutic approaches.

## Supporting information

Fang et al. supplementary material Fang et al. supplementary material

## Supplementary material

The supplementary material for this article can be found at http://doi.org/10.1017/S0033291725101499.
