# Supplementary material for: Two subtypes of major depressive disorder are identified from individualized gray matter morphological abnormalities in a large multi-site dataset
Source: Psychol Med. 2025 Sep 1;55:e257. doi: 10.1017/S0033291725101499 (PMC13054913; doi:10.1017/S0033291725101499)

**Table S1**. Gray matter morphological abnormalities of the identified subtypes and that of all patients relative to healthy controls.

| Subtypes | Clusters | Voxel number | Peak MNI | T | Including regions |
| --- | --- | --- | --- | --- | --- |
| Subtype 1 | 1 | 29218 | -1.5, -60, -51 | -9.25 | Cerebellum posterior lobe |
|  | 2 | 234 | -60, 3, -31.5 | 6.46 | Middle temporal gyrus |
|  | 3 | 3237 | 48, 45, -16.5 | 7.80 | Superior frontal gyrus |
|  |  |  |  |  | Inferior frontal gyrus |
|  |  |  |  |  | Middle frontal gyrus |
|  | 4 | 4065 | -12, 52.5, -6 | 9.71 | Superior frontal gyrus |
|  |  |  |  |  | Medial frontal gyrus |
|  | 5 | 504 | 64.5, -54, -19.5 | 6.92 | Inferior temporal gyrus |
|  | 6 | 296 | -37.5, 51, -18 | 6.13 | Middle frontal gyrus |
|  | 7 | 783 | 13.5, 52.5, 0 | 7.07 | Medial frontal gyrus |
|  | 8 | 1180 | -37.5, -94.5, -6 | 6.52 | Middle occipital gyrus |
|  | 9 | 405 | -58.5, 27, 16.5 | 7.26 | Inferior frontal gyurs |
|  | 10 | 178 | 21, 22.5, 3 | 6.10 | Caudate |
|  | 11 | 1831 | 45, -31.5, 30 | 8.29 | Inferior parietal lobule |
|  |  |  |  |  | Insula |
|  |  |  |  |  | Superior temporal gyrus |
|  | 12 | 2256 | -37.5, -28.5, 1.5 | 7.73 | Insula |
|  |  |  |  |  | Superior temporal gyrus |
|  |  |  |  |  | Postcentral gyrus |
|  | 13 | 257 | 48, 1.5, 13.5 | 6.80 | Insula |
|  | 14 | 160 | -37.5, 15, 15 | 7.98 | Inferior frontal gyrus |
|  | 15 | 173 | 15, 40.5, 15 | 5.84 | Anterior cingulate cortex |
|  | 16 | 328 | -12, -72, 18 | 7.38 | Precuneus |
|  | 17 | 314 | -9, 36, 37.5 | 7.11 | Medial frontal gyrus |
|  | 18 | 153 | -37.5, 31.5, 45 | 6.43 | Middle frontal gyrus |
|  | 19 | 1058 | -4.5, -10.5, 78 | 8.82 | Superior frontal gyrus |
|  |  |  |  |  | Postcentral gyrus |
|  |  |  |  |  | Precentral gyrus |
| Subtype 2 | 1 | 2949 | -36, -37.5, -51 | 7.99 | Cerebellum posterior lobe |
|  | 2 | 3551 | -10.5, -58.5, -42 | 8.35 | Cerebellar tonsil |
|  | 3 | 110533 | -9, 13.5, 27 | -10.20 | Frontal gyrus |
|  |  |  |  |  | Insula |
|  |  |  |  |  | Precuneus |
|  |  |  |  |  | Temporal gyrus |
|  |  |  |  |  | Striatum |
|  | 4 | 250 | -49.5, -70.5, -36 | 6.29 | Cerebellum posterior lobe |
|  | 5 | 325 | -21, -73.5, -30 | 5.96 | Cerebellum posterior lobe |
|  | 6 | 177 | -28.5, -28.5, -4.5 | -5.89 | Hippcampus |
|  | 7 | 295 | -7.5, 16.5, 10.5 | -6.77 | Caudate |
|  | 8 | 334 | -31.5, -70.5, 36 | -6.06 | Middle occipital gyrus |
|  | 9 | 353 | 435.5, -31.5, 34.5 | -8.53 | Inferior parietal lobule |
|  | 10 | 382 | 33, -27, 51 | -7.26 | Precentral gyrus |
| All | 1 | 934 | -43.5, 13.5, -3 | -7.40 | Superior temporal gyrus |
|  | 2 | 202 | 69, -51, -16.5 | 6.69 | Inferior temporal gyrus |
|  | 3 | 345 | -20, 27, -9 | -6.70 | Inferior frontal gyrus |
|  |  |  |  |  | Insula |
|  | 4 | 200 | 49.5, 13.5, -4.5 | -6.87 | Superior temporal gyrus |
|  | 5 | 216 | -7.5, 16.5, 10.5 | -6.88 | Caudate |
|  | 6 | 2513 | -9, 12, 27 | -8.74 | Cingulate gyrus |
|  |  |  |  |  | Caudate |
|  | 7 | 263 | 42, -33, 34.5 | -8.04 | Inferior parietal lobule |
|  | 8 | 573 | 12, -19.5, 34.5 | -7.59 | Cingulate gyrus |
|  | 9 | 209 | 15, 37.5, 37.5 | -7.65 | Superior frontal gyrus |
|  | 10 | 355 | -25.5, -16.5, 64.5 | -6.49 | Precentral gyrus |
|  | 11 | 440 | -4.5, -10.5, 78 | 8.61 | Superior frontal gyrus |

**Figure S1**. The performance of Gaussian process regression in inferring GMVs in healthy controls. The average standardized mean squared error (MSE) values between true GMVs and predicted ones across 100 runs of 10–fold cross-validation and leave-one-site-out cross-validation are mapped on the brain.


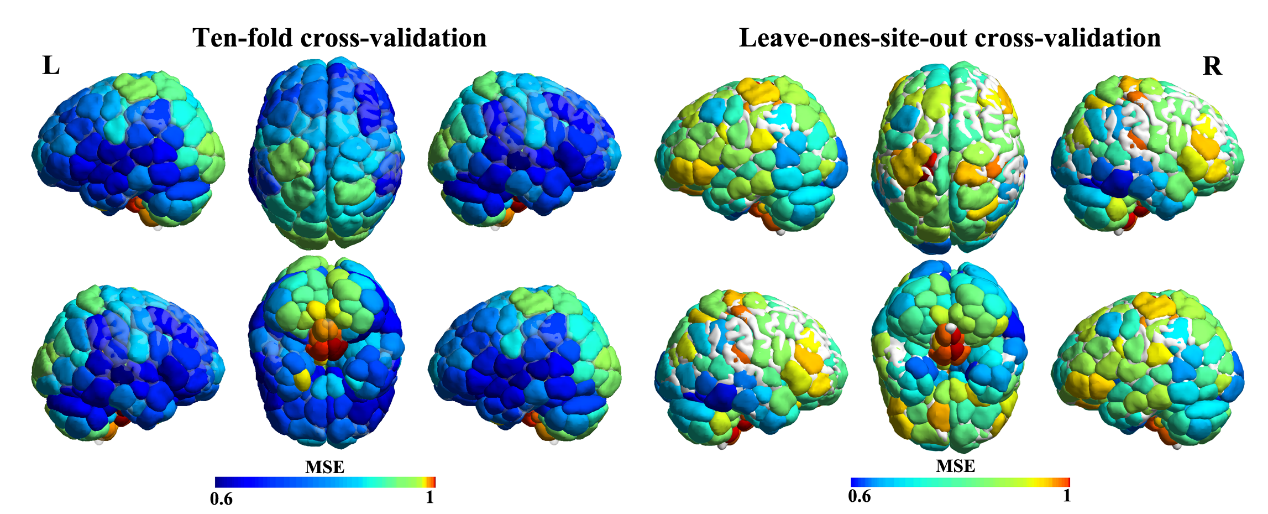


**Figure S2**. Statistical significance of clustering using Shen 268 brain atlas. The dash-line indicates the cluster index derived from the k-means clustering, calculated as the ratio of the sum of within-cluster sums of squares to the total sum of squares around the overall mean. The P-value represents the proportion of simulated cluster indices that exceed the empirical P-value. P-vNorm refers to the P-value obtained from a Gaussian fit.


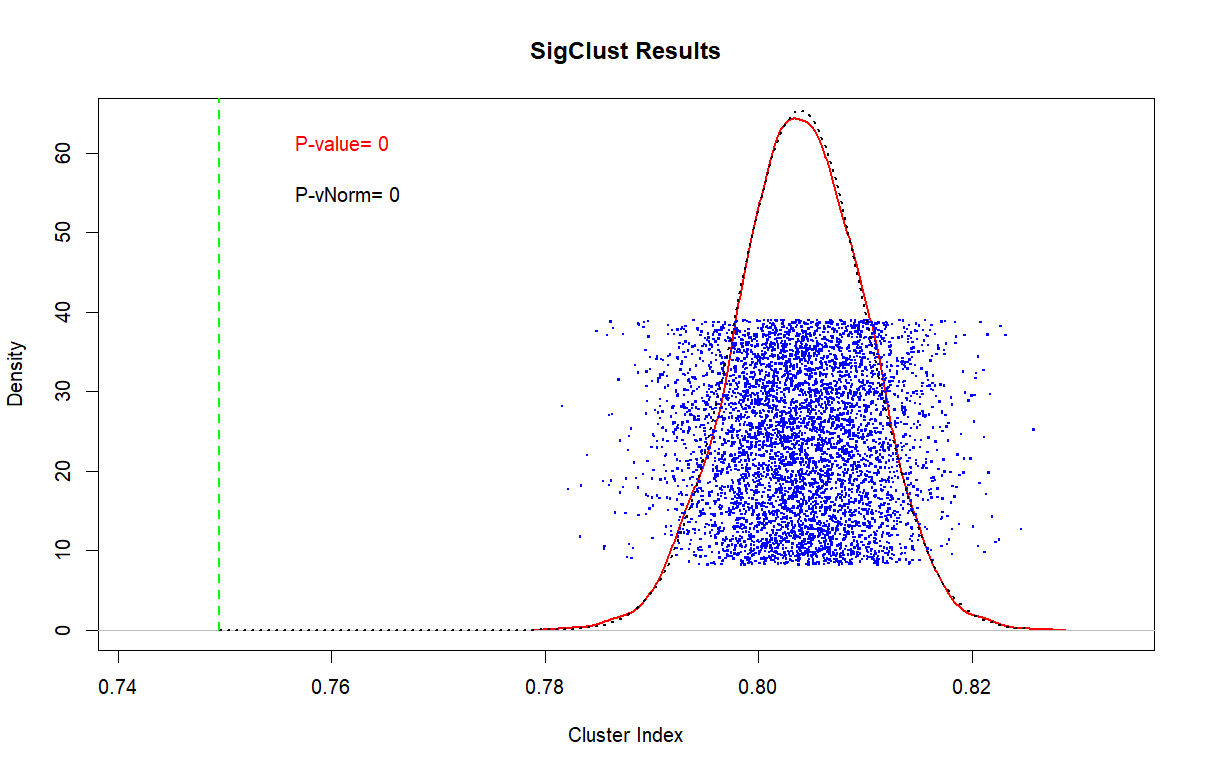


**Figure S3**. Statistical significance of clustering using Automated Anatomical Labeling (AAL) atlas. The dash-line indicates the cluster index derived from the k-means clustering, calculated as the ratio of the sum of within-cluster sums of squares to the total sum of squares around the overall mean. The P-value represents the proportion of simulated cluster indices that exceed the empirical P-value. P-vNorm refers to the P-value obtained from a Gaussian fit.


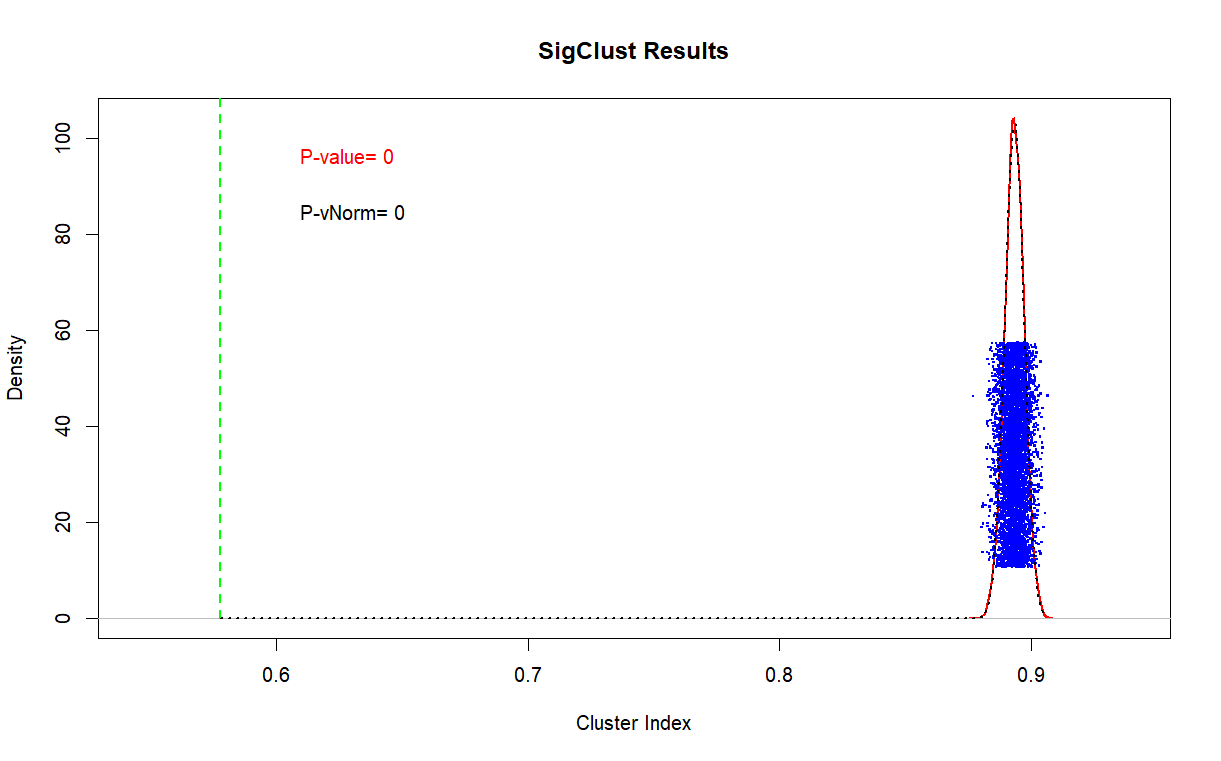


**Figure S4**. Subtyping results using the Automated Anatomical Labeling (AAL) atlas. (A) The optimal two-cluster solution identified by the cluster ensemble voting technique (marked by red asterisk). (B) Voxel-wise gray matter abnormalities in the identified subtypes relative to healthy controls.


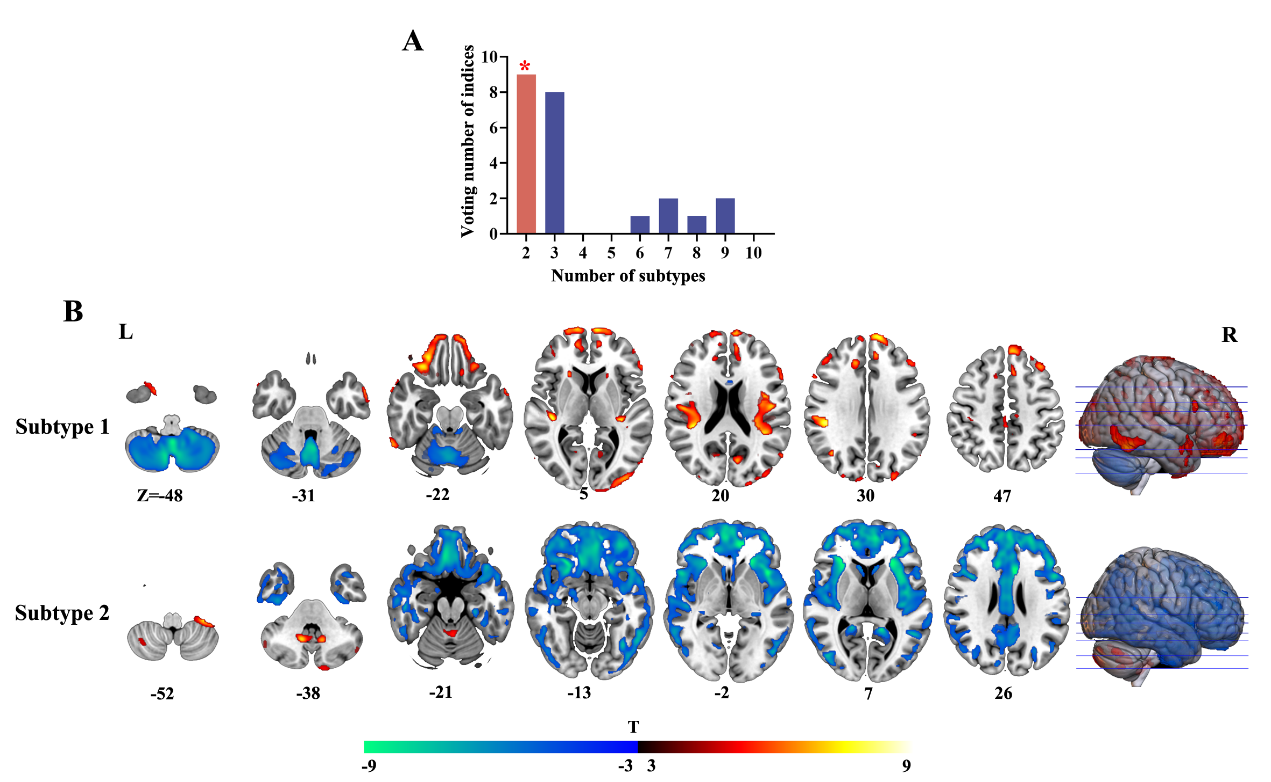

Supplement: Fang et al. supplementary material [file S0033291725101499sup001.docx]
